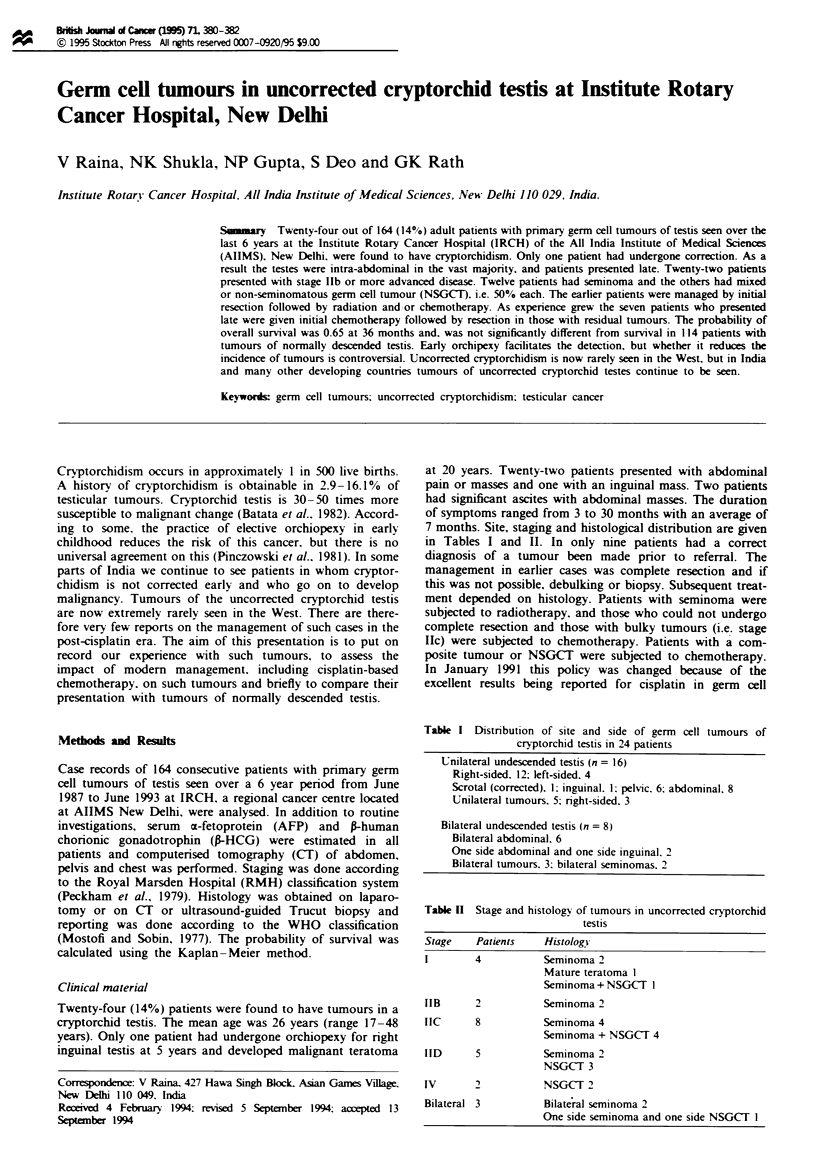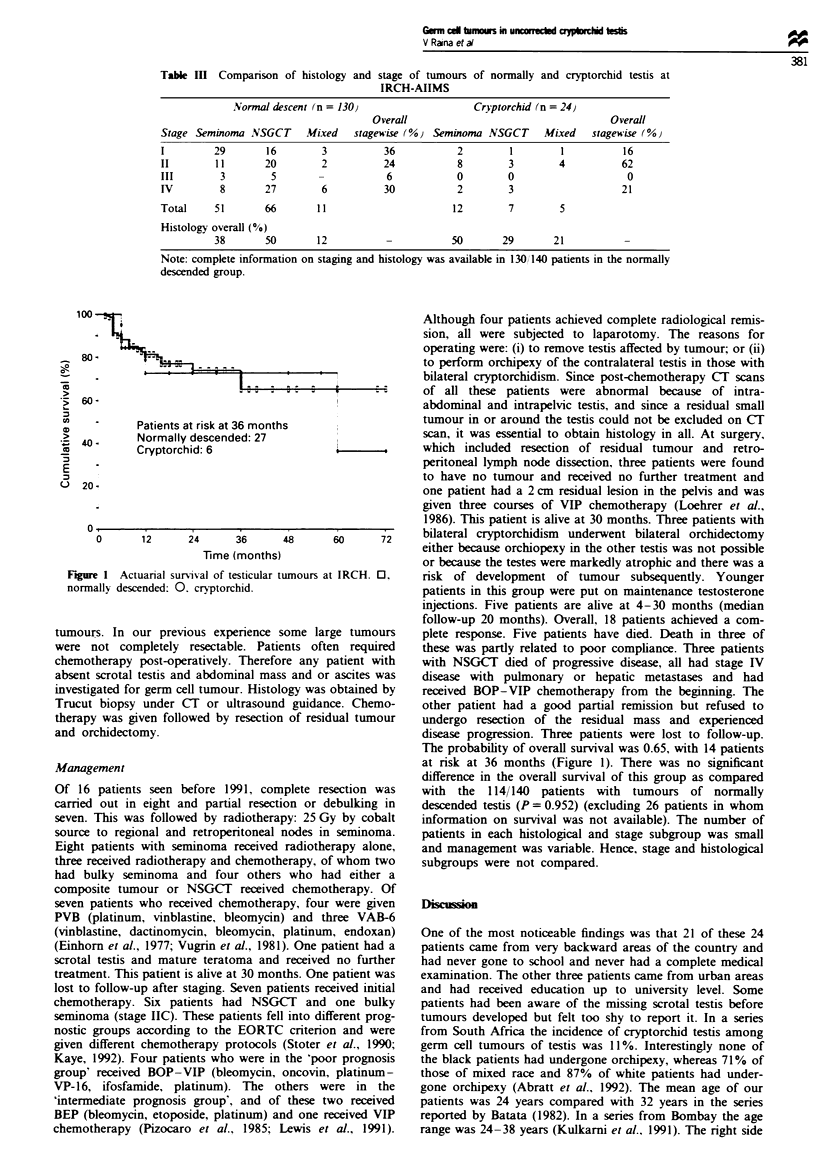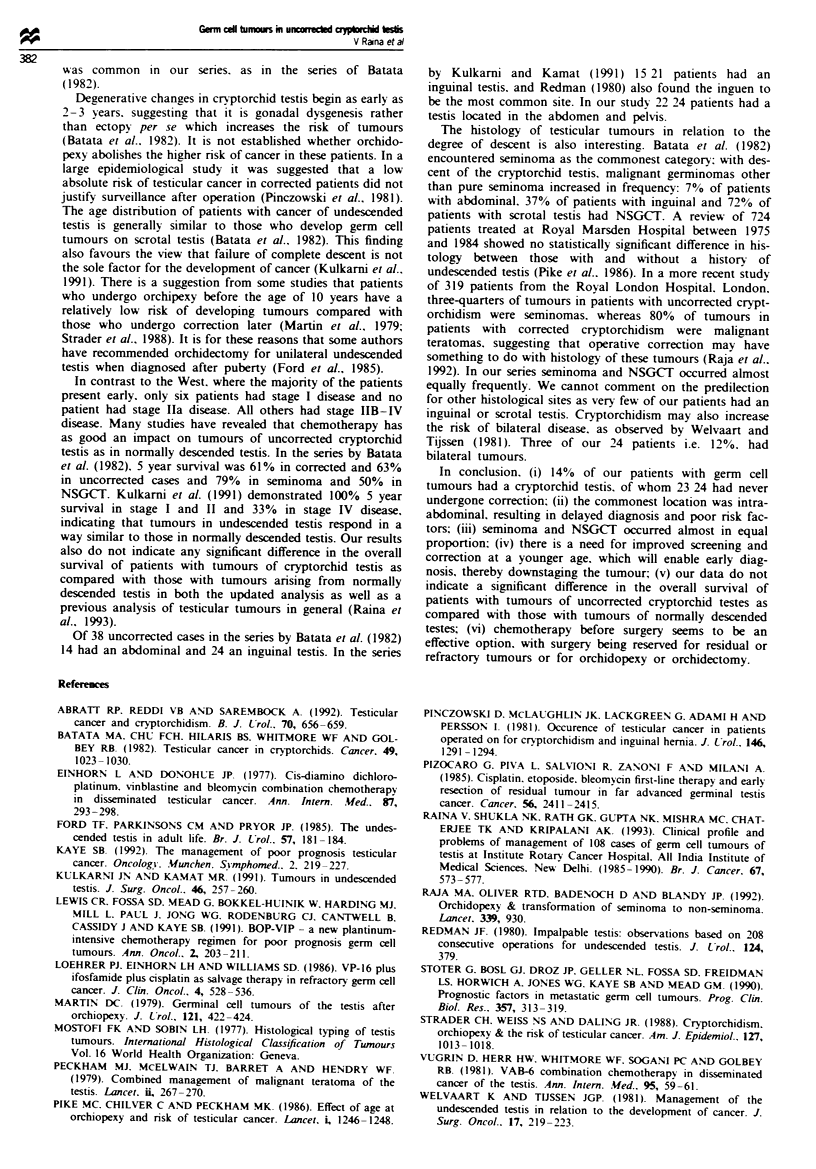# Germ cell tumours in uncorrected cryptorchid testis at Institute Rotary Cancer Hospital, New Delhi.

**DOI:** 10.1038/bjc.1995.77

**Published:** 1995-02

**Authors:** V. Raina, N. K. Shukla, N. P. Gupta, S. Deo, G. K. Rath

**Affiliations:** Institute Rotary Cancer Hospital, All India Institute of Medical Sciences, New Delhi.

## Abstract

Twenty-four out of 164 (14%) adult patients with primary germ cell tumours of testis seen over the last 6 years at the Institute Rotary Cancer Hospital (IRCH) of the All India Institute of Medical Sciences (AIIMS), New Delhi, were found to have cryptorchidism. Only one patient had undergone correction. As a result the testes were intra-abdominal in the vast majority, and patients presented late. Twenty-two patients presented with stage IIb or more advanced disease. Twelve patients had seminoma and the others had mixed or non-seminomatous germ cell tumour (NSGCT), i.e. 50% each. The earlier patients were managed by initial resection followed by radiation and/or chemotherapy. As experience grew the seven patients who presented late were given initial chemotherapy followed by resection in those with residual tumours. The probability of overall survival was 0.65 at 36 months and, was not significantly different from survival in 114 patients with tumours of normally descended testis. Early orchipexy facilitates the detection, but whether it reduces the incidence of tumours is controversial. Uncorrected cryptorchidism is now rarely seen in the West, but in India and many other developing countries tumours of uncorrected cryptorchid testes continue to be seen.


British Journal of Cancer (1995) 71, 380-382

© 1995 Stockton Press All rights reserved 0007-0920/95 $9.00

### Germ cell tumours in uncorrected cryptorchid testis at Institute Rotary Cancer Hospital, New Delhi

V Raina, NK Shukla, NP Gupta, S Deo and GK Rath

*Institute Rotary Cancer Hospital, All India Institute of Medical Sciences, New Delhi 110 029, India.*

**Summary** Twenty-four out of 164 (14%) adult patients with primary germ cell tumours of testis seen over the last 6 years at the Institute Rotary Cancer Hospital (IRCH) of the All India Institute of Medical Sciences (AIIMS), New Delhi, were found to have cryptorchidism. Only one patient had undergone correction. As a result the testes were intra-abdominal in the vast majority, and patients presented late. Twenty-two patients presented with stage IIb or more advanced disease. Twelve patients had seminoma and the others had mixed or non-seminomatous germ cell tumour (NSGCT), i.e. 50% each. The earlier patients were managed by initial resection followed by radiation and or chemotherapy. As experience grew the seven patients who presented late were given initial chemotherapy followed by resection in those with residual tumours. The probability of overall survival was 0.65 at 36 months and, was not significantly different from survival in 114 patients with tumours of normally descended testis. Early orchipexy facilitates the detection, but whether it reduces the incidence of tumours is controversial. Uncorrected cryptorchidism is now rarely seen in the West, but in India and many other developing countries tumours of uncorrected cryptorchid testes continue to be seen.

**Keywords:** germ cell tumours; uncorrected cryptorchidism; testicular cancer

Cryptorchidism occurs in approximately 1 in 500 live births. A history of cryptorchidism is obtainable in 2.9-16.1% of testicular tumours. Cryptorchid testis is 30-50 times more susceptible to malignant change (Batata *et al.*, 1982). According to some, the practice of elective orchiopexy in early childhood reduces the risk of this cancer, but there is no universal agreement on this (Pinczowski *et al.*, 1981). In some parts of India we continue to see patients in whom cryptorchidism is not corrected early and who go on to develop malignancy. Tumours of the uncorrected cryptorchid testis are now extremely rarely seen in the West. There are therefore very few reports on the management of such cases in the post-cisplatin era. The aim of this presentation is to put on record our experience with such tumours, to assess the impact of modern management, including cisplatin-based chemotherapy, on such tumours and briefly to compare their presentation with tumours of normally descended testis.

### Methods and Results

Case records of 164 consecutive patients with primary germ cell tumours of testis seen over a 6 year period from June 1987 to June 1993 at IRCH, a regional cancer centre located at AIIMS New Delhi, were analysed. In addition to routine investigations, serum α-fetoprotein (AFP) and β-human chorionic gonadotrophin (β-HCG) were estimated in all patients and computerised tomography (CT) of abdomen, pelvis and chest was performed. Staging was done according to the Royal Marsden Hospital (RMH) classification system (Peckham *et al.*, 1979). Histology was obtained on laparotomy or on CT or ultrasound-guided Trucut biopsy and reporting was done according to the WHO classification (Mostofi and Sobin, 1977). The probability of survival was calculated using the Kaplan-Meier method.

### *Clinical material*

Twenty-four (14%) patients were found to have tumours in a cryptorchid testis. The mean age was 26 years (range 17-48 years). Only one patient had undergone orchiopexy for right inguinal testis at 5 years and developed malignant teratoma at 20 years. Twenty-two patients presented with abdominal pain or masses and one with an inguinal mass. Two patients had significant ascites with abdominal masses. The duration of symptoms ranged from 3 to 30 months with an average of 7 months. Site, staging and histological distribution are given in Tables I and II. In only nine patients had a correct diagnosis of a tumour been made prior to referral. The management in earlier cases was complete resection and if this was not possible, debulking or biopsy. Subsequent treatment depended on histology. Patients with seminoma were subjected to radiotherapy, and those who could not undergo complete resection and those with bulky tumours (i.e. stage IIc) were subjected to chemotherapy. Patients with a composite tumour or NSGCT were subjected to chemotherapy. In January 1991 this policy was changed because of the excellent results being reported for cisplatin in germ cell

Correspondence: V Raina, 427 Hawa Singh Block, Asian Games Village, New Delhi 110 049, India
Received 4 February 1994; revised 5 September 1994; accepted 13 September 1994

**Table I** Distribution of site and side of germ cell tumours of cryptorchid testis in 24 patients

| |
|---|
| Unilateral undescended testis (*n* = 16) |
| Right-sided, 12; left-sided, 4 |
| Scrotal (corrected), 1; inguinal, 1; pelvic, 6; abdominal, 8 |
| Unilateral tumours, 5; right-sided, 3 |
| Bilateral undescended testis (*n* = 8) |
| Bilateral abdominal, 6 |
| One side abdominal and one side inguinal, 2 |
| Bilateral tumours, 3; bilateral seminomas, 2 |

**Table II** Stage and histology of tumours in uncorrected cryptorchid testis

| *Stage* | *Patients* | *Histology* |
|---|---|---|
| I | 4 | Seminoma 2 |
| | | Mature teratoma 1 |
| | | Seminoma + NSGCT 1 |
| IIB | 2 | Seminoma 2 |
| IIC | 8 | Seminoma 4 |
| | | Seminoma + NSGCT 4 |
| IID | 5 | Seminoma 2 |
| | | NSGCT 3 |
| IV | 2 | NSGCT 2 |
| Bilateral | 3 | Bilateral seminoma 2 |
| | | One side seminoma and one side NSGCT 1 |

**Table III** Comparison of histology and stage of tumours of normally and cryptorchid testis at IRCH-AIIMS

| | *Normal descent* (n = *130*) | | | | *Cryptorchid* (n = *24*) | | | |
|---|---|---|---|---|---|---|---|---|
| *Stage* | *Seminoma* | *NSGCT* | *Mixed* | *Overall stagewise* (*%*) | *Seminoma* | *NSGCT* | *Mixed* | *Overall stagewise* (*%*) |
| I | 29 | 16 | 3 | 36 | 2 | 1 | 1 | 16 |
| II | 11 | 20 | 2 | 24 | 8 | 3 | 4 | 62 |
| III | 3 | 5 | – | 6 | 0 | 0 | | 0 |
| IV | 8 | 27 | 6 | 30 | 2 | 3 | | 21 |
| Total | 51 | 66 | 11 | | 12 | 7 | 5 | |
| Histology overall (%) | | | | | | | | |
| | 38 | 50 | 12 | – | 50 | 29 | 21 | – |

Note: complete information on staging and histology was available in 130/140 patients in the normally descended group.

**Figure 1** Actuarial survival of testicular tumours at IRCH. □, normally descended; O, cryptorchid.

tumours. In our previous experience some large tumours were not completely resectable. Patients often required chemotherapy post-operatively. Therefore any patient with absent scrotal testis and abdominal mass and or ascites was investigated for germ cell tumour. Histology was obtained by Trucut biopsy under CT or ultrasound guidance. Chemotherapy was given followed by resection of residual tumour and orchidectomy.

### *Management*

Of 16 patients seen before 1991, complete resection was carried out in eight and partial resection or debulking in seven. This was followed by radiotherapy: 25 Gy by cobalt source to regional and retroperitoneal nodes in seminoma. Eight patients with seminoma received radiotherapy alone, three received radiotherapy and chemotherapy, of whom two had bulky seminoma and four others who had either a composite tumour or NSGCT received chemotherapy. Of seven patients who received chemotherapy, four were given PVB (platinum, vinblastine, bleomycin) and three VAB-6 (vinblastine, dactinomycin, bleomycin, platinum, endoxan) (Einhorn *et al.*, 1977; Vugrin *et al.*, 1981). One patient had a scrotal testis and mature teratoma and received no further treatment. This patient is alive at 30 months. One patient was lost to follow-up after staging. Seven patients received initial chemotherapy. Six patients had NSGCT and one bulky seminoma (stage IIC). These patients fell into different prognostic groups according to the EORTC criterion and were given different chemotherapy protocols (Stoter *et al.*, 1990; Kaye, 1992). Four patients who were in the 'poor prognosis group' received BOP–VIP (bleomycin, oncovin, platinum–VP-16, ifosfamide, platinum). The others were in the 'intermediate prognosis group', and of these two received BEP (bleomycin, etoposide, platinum) and one received VIP chemotherapy (Pizocaro *et al.*, 1985; Lewis *et al.*, 1991).

Although four patients achieved complete radiological remission, all were subjected to laparotomy. The reasons for operating were: (i) to remove testis affected by tumour; or (ii) to perform orchipexy of the contralateral testis in those with bilateral cryptorchidism. Since post-chemotherapy CT scans of all these patients were abnormal because of intra-abdominal and intrapelvic testis, and since a residual small tumour in or around the testis could not be excluded on CT scan, it was essential to obtain histology in all. At surgery, which included resection of residual tumour and retroperitoneal lymph node dissection, three patients were found to have no tumour and received no further treatment and one patient had a 2 cm residual lesion in the pelvis and was given three courses of VIP chemotherapy (Loehrer *et al.*, 1986). This patient is alive at 30 months. Three patients with bilateral cryptorchidism underwent bilateral orchidectomy either because orchiopexy in the other testis was not possible or because the testes were markedly atrophic and there was a risk of development of tumour subsequently. Younger patients in this group were put on maintenance testosterone injections. Five patients are alive at 4–30 months (median follow-up 20 months). Overall, 18 patients achieved a complete response. Five patients have died. Death in three of these was partly related to poor compliance. Three patients with NSGCT died of progressive disease, all had stage IV disease with pulmonary or hepatic metastases and had received BOP–VIP chemotherapy from the beginning. The other patient had a good partial remission but refused to undergo resection of the residual mass and experienced disease progression. Three patients were lost to follow-up. The probability of overall survival was 0.65, with 14 patients at risk at 36 months (Figure 1). There was no significant difference in the overall survival of this group as compared with the 114/140 patients with tumours of normally descended testis ($P = 0.952$) (excluding 26 patients in whom information on survival was not available). The number of patients in each histological and stage subgroup was small and management was variable. Hence, stage and histological subgroups were not compared.

### Discussion

One of the most noticeable findings was that 21 of these 24 patients came from very backward areas of the country and had never gone to school and never had a complete medical examination. The other three patients came from urban areas and had received education up to university level. Some patients had been aware of the missing scrotal testis before tumours developed but felt too shy to report it. In a series from South Africa the incidence of cryptorchid testis among germ cell tumours of testis was 11%. Interestingly none of the black patients had undergone orchipexy, whereas 71% of those of mixed race and 87% of white patients had undergone orchipexy (Abratt *et al.*, 1992). The mean age of our patients was 24 years compared with 32 years in the series reported by Batata (1982). In a series from Bombay the age range was 24–38 years (Kulkarni *et al.*, 1991). The right side was common in our series, as in the series of Batata (1982).

Degenerative changes in cryptorchid testis begin as early as 2–3 years, suggesting that it is gonadal dysgenesis rather than ectopy *per se* which increases the risk of tumours (Batata *et al.*, 1982). It is not established whether orchidopexy abolishes the higher risk of cancer in these patients. In a large epidemiological study it was suggested that a low absolute risk of testicular cancer in corrected patients did not justify surveillance after operation (Pinczowski *et al.*, 1981). The age distribution of patients with cancer of undescended testis is generally similar to those who develop germ cell tumours on scrotal testis (Batata *et al.*, 1982). This finding also favours the view that failure of complete descent is not the sole factor for the development of cancer (Kulkarni *et al.*, 1991). There is a suggestion from some studies that patients who undergo orchipexy before the age of 10 years have a relatively low risk of developing tumours compared with those who undergo correction later (Martin *et al.*, 1979; Strader *et al.*, 1988). It is for these reasons that some authors have recommended orchidectomy for unilateral undescended testis when diagnosed after puberty (Ford *et al.*, 1985).

In contrast to the West, where the majority of the patients present early, only six patients had stage I disease and no patient had stage IIa disease. All others had stage IIB–IV disease. Many studies have revealed that chemotherapy has as good an impact on tumours of uncorrected cryptorchid testis as in normally descended testis. In the series by Batata *et al.* (1982), 5 year survival was 61% in corrected and 63% in uncorrected cases and 79% in seminoma and 50% in NSGCT. Kulkarni *et al.* (1991) demonstrated 100% 5 year survival in stage I and II and 33% in stage IV disease, indicating that tumours in undescended testis respond in a way similar to those in normally descended testis. Our results also do not indicate any significant difference in the overall survival of patients with tumours of cryptorchid testis as compared with those with tumours arising from normally descended testis in both the updated analysis as well as a previous analysis of testicular tumours in general (Raina *et al.*, 1993).

Of 38 uncorrected cases in the series by Batata *et al.* (1982) 14 had an abdominal and 24 an inguinal testis. In the series by Kulkarni and Kamat (1991) 15 21 patients had an inguinal testis, and Redman (1980) also found the inguen to be the most common site. In our study 22 24 patients had a testis located in the abdomen and pelvis.

The histology of testicular tumours in relation to the degree of descent is also interesting. Batata *et al.* (1982) encountered seminoma as the commonest category; with descent of the cryptorchid testis, malignant germinomas other than pure seminoma increased in frequency: 7% of patients with abdominal, 37% of patients with inguinal and 72% of patients with scrotal testis had NSGCT. A review of 724 patients treated at Royal Marsden Hospital between 1975 and 1984 showed no statistically significant difference in histology between those with and without a history of undescended testis (Pike *et al.*, 1986). In a more recent study of 319 patients from the Royal London Hospital, London, three-quarters of tumours in patients with uncorrected cryptorchidism were seminomas, whereas 80% of tumours in patients with corrected cryptorchidism were malignant teratomas, suggesting that operative correction may have something to do with histology of these tumours (Raja *et al.*, 1992). In our series seminoma and NSGCT occurred almost equally frequently. We cannot comment on the predilection for other histological sites as very few of our patients had an inguinal or scrotal testis. Cryptorchidism may also increase the risk of bilateral disease, as observed by Welvaart and Tijssen (1981). Three of our 24 patients i.e. 12%, had bilateral tumours.

In conclusion, (i) 14% of our patients with germ cell tumours had a cryptorchid testis, of whom 23 24 had never undergone correction; (ii) the commonest location was intra-abdominal, resulting in delayed diagnosis and poor risk factors; (iii) seminoma and NSGCT occurred almost in equal proportion; (iv) there is a need for improved screening and correction at a younger age, which will enable early diagnosis, thereby downstaging the tumour; (v) our data do not indicate a significant difference in the overall survival of patients with tumours of uncorrected cryptorchid testes as compared with those with tumours of normally descended testes; (vi) chemotherapy before surgery seems to be an effective option, with surgery being reserved for residual or refractory tumours or for orchidopexy or orchidectomy.

### References

ABRATT RP, REDDI VB AND SAREMBOCK A. (1992). Testicular cancer and cryptorchidism. *B. J. Urol.*, **70**, 656–659.

BATATA MA, CHU FCH, HILARIS BS, WHITMORE WF AND GOLBEY RB. (1982). Testicular cancer in cryptorchids. *Cancer*, **49**, 1023–1030.

EINHORN L AND DONOHUE JP. (1977). Cis-diamino dichloroplatinum, vinblastine and bleomycin combination chemotherapy in disseminated testicular cancer. *Ann. Intern. Med.*, **87**, 293–298.

FORD TF, PARKINSONS CM AND PRYOR JP. (1985). The undescended testis in adult life. *Br. J. Urol.*, **57**, 181–184.

KAYE SB. (1992). The management of poor prognosis testicular cancer. *Oncology, Munchen, Symphomed.*, 2, 219–227.

KULKARNI JN AND KAMAT MR. (1991). Tumours in undescended testis. *J. Surg. Oncol.*, **46**, 257–260.

LEWIS CR, FOSSA SD, MEAD G, BOKKEL-HUINIK W, HARDING MJ, MILL L, PAUL J, JONG WG, RODENBURG CJ, CANTWELL B, CASSIDY J AND KAYE SB. (1991). BOP-VIP – a new plantinum-intensive chemotherapy regimen for poor prognosis germ cell tumours. *Ann. Oncol.*, **2**, 203–211.

LOEHRER PJ, EINHORN LH AND WILLIAMS SD. (1986). VP-16 plus ifosfamide plus cisplatin as salvage therapy in refractory germ cell cancer. *J. Clin. Oncol.*, **4**, 528–536.

MARTIN DC. (1979). Germinal cell tumours of the testis after orchiopexy. *J. Urol.*, **121**, 422–424.

MOSTOFI FK AND SOBIN LH. (1977). Histological typing of testis tumours. *International Histological Classification of Tumours* Vol. 16 World Health Organization: Geneva.

PECKHAM MJ, MCELWAIN TJ, BARRET A AND HENDRY WF. (1979). Combined management of malignant teratoma of the testis. *Lancet*, **ii**, 267–270.

PIKE MC, CHILVER C AND PECKHAM MK. (1986). Effect of age at orchiopexy and risk of testicular cancer. *Lancet*, **i**, 1246–1248.

PINCZOWSKI D, MCLAUGHLIN JK, LACKGREEN G, ADAMI H AND PERSSON I. (1981). Occurence of testicular cancer in patients operated on for cryptorchidism and inguinal hernia. *J. Urol.*, **146**, 1291–1294.

PIZOCARO G, PIVA L, SALVIONI R, ZANONI F AND MILANI A. (1985). Cisplatin, etoposide, bleomycin first-line therapy and early resection of residual tumour in far advanced germinal testis cancer. *Cancer*, **56**, 2411–2415.

RAINA V, SHUKLA NK, RATH GK, GUPTA NK, MISHRA MC, CHATERJEE TK AND KRIPALANI AK. (1993). Clinical profile and problems of management of 108 cases of germ cell tumours of testis at Institute Rotary Cancer Hospital, All India Institute of Medical Sciences, New Delhi, (1985–1990). *Br. J. Cancer*, **67**, 573–577.

RAJA MA, OLIVER RTD, BADENOCH D AND BLANDY JP. (1992). Orchidopexy & transformation of seminoma to non-seminoma. *Lancet*, **339**, 930.

REDMAN JF. (1980). Impalpable testis: observations based on 208 consecutive operations for undescended testis. *J. Urol.*, **124**, 379.

STOTER G, BOSL GJ, DROZ JP, GELLER NL, FOSSA SD, FREIDMAN LS, HORWICH A, JONES WG, KAYE SB AND MEAD GM. (1990). Prognostic factors in metastatic germ cell tumours. *Prog. Clin. Biol. Res.*, **357**, 313–319.

STRADER CH, WEISS NS AND DALING JR. (1988). Cryptorchidism, orchiopexy & the risk of testicular cancer. *Am. J. Epidemiol.*, **127**, 1013–1018.

VUGRIN D, HERR HW, WHITMORE WF, SOGANI PC AND GOLBEY RB. (1981). VAB-6 combination chemotherapy in disseminated cancer of the testis. *Ann. Intern. Med.*, **95**, 59–61.

WELVAART K AND TIJSSEN JGP. (1981). Management of the undescended testis in relation to the development of cancer. *J. Surg. Oncol.*, **17**, 219–223.